# Gene-based molecular characterization of *cox1* and *pnad5* in *Hymenolepis nana* isolated from naturally infected mice and rats in Saudi Arabia

**DOI:** 10.1042/BSR20181224

**Published:** 2019-02-08

**Authors:** Dina M. Metwally, Huda A. Al-Enezy, Isra M. Al-Turaiki, Manal F. El-Khadragy, Hany M. Yehia, Tahani T. Al-Otaibi

**Affiliations:** 1 Department of Zoology, Faculty of Science, King Saud University, Riyadh, Saudi Arabia; 2 Department of Parasitology, Faculty of Veterinary Medicine, Zagazig University, Zagazig, Egypt; 3 Department of Information Technology, Faculty of Computer and Information Science, King Saud University, Riyadh, Saudi Arabia; 4 Department of Zoology and Entomology, Faculty of Science, Helwan University, Cairo, Egypt; 5 Department of Food Science and Nutrition, Faculty of Food and Agriculture Sciences, King Saud University, Riyadh, Saudi Arabia; 6 Department of Food Science and Nutrition, Faculty of Home Economics, Helwan University, Cairo, Egypt; 7 Department of Biology, Faculty of Arts and Sciences, Nairiyah, Hafr Al-Batin University, Saudi Arabia

**Keywords:** Hymenolepis nana, Mitochondrial genome, Phylogenetic analysis

## Abstract

Mice and rats are animals commonly used in research and laboratory testing. Compared with other animal species, they harbor many more zoonotic agents. *Hymenolepis nana* (*H. nana*) is a common tapeworm that parasitizes both humans and rodents. Although this tapeworm is of socio-economic importance worldwide, information related to its mitochondrial genome is limited. The present study examined the sequence diversity of two mitochondrial (mt) genes, subunit I of cytochrome oxidase (*cox1*) and NADH dehydrogenase subunit 5 (*pnad5*), of *H. nana* in mice and rats from two geographical regions of Saudi Arabia (Makkah and Riyadh). Partial sequences of *cox1* and *pnad 5* from individual *H. nana* isolates were separately amplified using polymerase chain reaction (PCR) and sequenced. The GC contents of the sequences ranged between 31.6–33.5% and 27.2–28.6% for *cox1* and *pnad5*, respectively. The genomic similarity among specimens determined via *cox1* primer and *pnad5* primer was 97.1% and 99.7%, respectively. Based on these primers, our data did not indicate any differences between *H. nana* from rat and mice isolates. Results demonstrated that the present species are deeply embedded in the genus *Hymenolepis* with close relationship to other *Hymenolepis* species, including *H. nana* as a putative sister taxon, and that the isolates cannot be categorized as belonging to two different groups with origins in Makkah and Riyadh.

## Introduction

*Hymenolepis nana*, a common tapeworm that is distributed worldwide, is found mostly in young children in developing countries [1]. Human hymenolepiasis, caused by *H. nana* and *H. diminuta*, is a globally prevalent zoonosis. It is endemic in Asia, Southern and Eastern Europe, Central and South America, and Africa, and produces many health problems such as headaches, weakness, anorexia, abdominal pain, and diarrhea [2]. The mature worm lives in the small intestine of humans, mice, and rats [3]. It is mostly transmitted by contamination with fecal matter containing eggs or via insect vectors acting as intermediate hosts. *Hymenolepis nana*, is also able to complete its entire life cycle in a single host, and is therefore capable of auto-infection [4]. *Hymenolepis nana*, in different rodents, such as rats and mice, is morphologically similar to human *H. nana*. Thus, establishing the identity of these two species is epidemiologically important [5]. Despite revised nomenclature, speciation and host specificity of *H. nana* continues to be problematic [6]. Hence, a biological, taxonomic and epidemiological investigation of *H. nana* in various hosts may be useful in order to better understand endemic strains [6].

Hymenolepidids have been categorized into several genera based on morphological characteristics [7, 8]. Mitochondrial (mt) genomes are small (usually less than 20,000 bp), circular and maternally inherited [9]. The property of having a high copy number per cell makes them attractive and more amenable targets for studies related to characterization, population genetics, and phylogenetics [10]. Mitochondrial DNA (mtDNA) sequences are reliable genetic markers that have been useful in studies on population genetics and systematics [11]. Genetic diversity of *H. nana* has been studied using genetic makers, such as the mt cytochrome oxidase subunit 1(*cox1*) and the entire first and second internal transcribed spacer (ITS-1 and ITS-2) regions of nuclear ribosomal DNA (rDNA) [6, 12, 13]. These studies indicated the presence of genetic variation in *H. nana* from different domestic and wildlife host species, as well as from different areas, suggesting that *H. nana* comprises ‘cryptic’ species, which are morphologically identical but genetically distinct. Although mitochondrial (mt) genes, such as NADH dehydrogenase subunit 5 (*pnad5*), small subunit ribosomal RNA (*rrnS*) and ATPase subunit6 (*atp6*), of *H. nana* in mice from different geographical regions of China have been studied, information on the sequence variability in other mt genes of *H. nana* isolates, is rare [14].

The objective of the present study was to analyze *cox1* and *pnad5* in *H. nana* isolated from naturally infected mice and rats in Makkah and Riyadh, Saudi Arabia.

This work was based on my previous study, “Gene-based molecular analysis of *cox1* in Echinococcus granulosus cysts isolated from naturally infected livestock in Riyadh, Saudi Arabia,” which was a part of a major research project. This project is conducted by the Zoology Department, Faculty of Science, King Saud University. The project aims to analyze genetic sequences of different parasites that are found spread out over Saudi Arabia, in order to help differentiate between the genetic sequences of local parasites and parasites of other regions, both inside and outside Saudi Arabia. Such information is expected to facilitate the development of methods for the prevention and control of these parasites.

## Materials and methods

### Sample collection

During the period between March and April of 2017, a total of 100 BALB/c mice (50 from Makkah and 50 from Riyadh) and 120 *Rattusu norvegis* rats (70 from Makkah and 50 from Riyadh) were obtained from the Female Center for Scientific and Medical Colleges, Riyadh, Saudi Arabia. The animals were kept in wire-bottomed cages in a room under conditions of standard illumination with a 12-h light–dark cycle, at a temperature of 25 ± 1°C for 1 week, until the commencement of treatment. Animals were provided with tap water and a balanced diet ad libitum. Mice were killed via decapitation. Worms were collected and extracted from all mice and rats, washed with normal saline and examined under a microscope to determine the type of worm. Worms were stored at −20°C until molecular analysis. All experiments were conducted according to specifications of the animal ethics committee outlined by the University of Sattam Bin Abdulaziz University (IRB number: SAU-2017-LAB-523/PI), which also included the joint efforts of Parasitology Department, Sattam Bin Abdulaziz University, and the College of Science, King Saud University.

### DNA extraction

Worms obtained from mice and rats were washed with distilled water and ethanol before they were centrifuged. Genomic DNA (gDNA) was then extracted using a High Pure PCR Template Preparation Kit (Qiagen GmbH, Hilden, Germany Cat. No.51304). Amplification of *cox1* and *pnad5* was performed using specific primers (*cox1***:** F:5′ AGAGTGATCCGGTGATATGGTGA 3′ R:5′ ACCATTCACCCTTGGTATAAGCAGA 3′, *pnad5***:** F:5′ GAAGCGTTAATTATGGGTT 3′ R:5′ GATTACAAGTTGATAGAGCCC 3′) [14] in a 40 μl reaction mixture containing 8 μl of master mix, 25.6 μl of deoxynucleotides (dNTPs), 2.4 μl of primers, and 4 μl of DNA template. The PCR program consisted of an initial denaturation step at 94°C for 5 min followed by 40 cycles of denaturation at 94°C for 45 s, annealing at 50°C for 45 s, extension at 72°C for 10 min, and a final extension step at 72°C for 10 min. PCR products were analyzed via 1% agarose gel electrophoresis.

### DNA sequencing and phylogenetic analysis

PCR products of *cox1* and *pnad5* were purified and sequenced using both forward and reverse complements by Genetic Analyzer at the Central Lab of King Saud University. A multiple sequence alignment was generated for the samples using the ClustalW [15] algorithm with a gap opening penalty of 10 and a gap extension penalty of 1. All sequences were truncated slightly using an error probability method with a limit of 0.05 at both ends. A BLAST search was performed for each sequence to locate related sequences. A phylogenetic tree was generated using MrBayes 3.2.6 [16], a Bayesian inference algorithm. Bootstrap method was used for resampling with the number of replicates set to 1000.

## Results

### Amplification of *cox1* and *pnad5*

Partial PCR amplification of *cox1* and *pnad5* yielded the expected 800 bp fragments for all DNA samples from both mice and rats.

### Analysis of *Cox1*

The sequences of 25 samples, including those of 10 Makkah mice, 13 Riyadh mice and 2 Makkah rats, were analyzed. The final sequences were 776–793 nucleotides in length (Table 1). A BLAST search was performed for each sequence to locate related sequences. All samples except one showed a pairwise identity of 99–99.60% and a 62–100% coverage relative to the genome of *H. nana*, Japan, with the accession number LM402005. In addition, all samples showed a pairwise identity of 99.00–99.60% and a 62–100% coverage relative to *H. nana* mt genome with accession numbers LM403673 and AP017666. All samples showed a pairwise identity of 97.90–98.60% and a 62–100% coverage to *H. nana* mt genome with the accession number KT951722.

**Table 1:** Genetic sequences from the isolated *H. nana* with variable lengths and GC contents (*cox1*)

Name	Host location	Host species	%GC	Post-Trim	Length
1MR	Riyadh, Saudi Arabia	*Mus musculus*	31.80%	756	790
2MR	Riyadh, Saudi Arabia	*Mus musculus*	31.80%	757	786
3MM	Makkah, Saudi Arabia	*Mus musculus*	32.00%	754	785
8MM	Makkah, Saudi Arabia	*Mus musculus*	31.80%	773	789
11MR	Riyadh, Saudi Arabia	*Mus musculus*	31.70%	765	793
12MR	Riyadh, Saudi Arabia	*Mus musculus*	32.20%	760	786
13MM	Makkah, Saudi Arabia	*Mus musculus*	31.60%	754	787
14MM	Makkah, Saudi Arabia	*Mus musculus*	32.00%	762	791
15MM	Makkah, Saudi Arabia	*Mus musculus*	31.90%	767	789
15MR	Riyadh, Saudi Arabia	*Mus musculus*	31.90%	755	786
16MM	Makkah, Saudi Arabia	*Mus musculus*	32.30%	748	787
16MR	Riyadh, Saudi Arabia	*Mus musculus*	31.80%	756	786
18MR	Riyadh, Saudi Arabia	*Mus musculus*	32.00%	758	776
19MR	Riyadh, Saudi Arabia	*Mus musculus*	32.20%	757	777
20MM	Makkah, Saudi Arabia	*Mus musculus*	31.90%	761	791
20MR	Riyadh, Saudi Arabia	*Mus musculus*	32.00%	760	788
24MR	Riyadh, Saudi Arabia	*Mus musculus*	32.00%	759	785
26MR	Riyadh, Saudi Arabia	*Mus musculus*	31.90%	759	788
27MM	Makkah, Saudi Arabia	*Mus musculus*	31.60%	779	787
32MR	Riyadh, Saudi Arabia	*Mus musculus*	32.10%	757	787
36MR	Riyadh, Saudi Arabia	*Mus musculus*	32.20%	758	786
37MM	Makkah, Saudi Arabia	*Mus musculus*	31.90%	763	789
42MM	Makkah, Saudi Arabia	*Mus musculus*	31.80%	759	787
23RM	Makkah, Saudi Arabia	*Rattus norvegius*	33.50%	759	786
40RM	Makkah, Saudi Arabia	*Rattus norvegius*	33.40%	203	293

All samples showed a pairwise identity of 98.80–99.60% and a 62.60–86.48% coverage with *H. nana* mt *cox1* gene, encoding cytochrome *c* oxidase subunit 1, partial *cds*, isolate: *H. nana* with accession number LC063187.

All samples showed a pairwise identity of more than 97.60% and 54% coverage with *H. nana cox1* partial *cds*, mitochondrial with accession numbers GU433102, GU433103, and GU433104. All of the samples showed a pairwise identity of 98.80–99.60% and 62.60–74% coverage to *H. nana cox1* mitochondrial gene, partial *cds* with accession number AB033412.

The multiple sequence alignment of the 25 samples and related sequences retrieved from Genbank was generated. The sequence LM402005 was set as the reference sequence. We found the following one-nucleotide substitutions (SNP) transitions: T to C at position 9998 of the reference sequence in 25% of the samples; C to T at position 10264 of the reference sequence in 92% of the samples; G to A at position 10495 of the reference sequence in all samples; and A to C at position 10591 of the reference sequence in all samples. In addition, one insertion of T was found at position 10766 of the reference sequence in 80% of the samples. Finally, two deletions of A were observed at positions 10760 and 10004, in 31% and 55% of the samples respectively (note: the alignment is provided in FASTA and Nexus formats).

**Figure 1:**
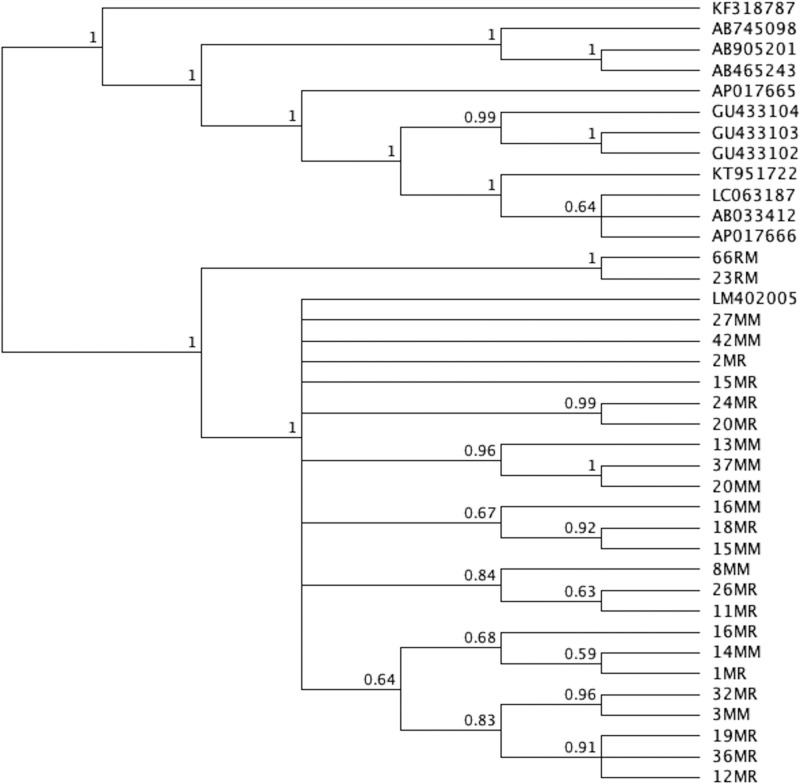
Phylogenetic tree of the 25 mice and rats samples in the present study along with similar sequences published in Genbank

The Phylogenetic tree was generated using MrBayes. *Dicrocoelium dendriticum* (Accession number KF318787) was used as the outgroup. The Phylogenetic tree with posterior probability values is shown (Figure 1). All the samples in the present study were grouped in a clade with *H. nana* genome assembly *H. nana*_Japan with accession number LM402005 (note: the tree is provided in Nexus format).

### Analysis of *pnad5*

Sequences of 31 samples, including, 10 Makkah mice, 17 Riyadh mice and 4 Makkah rats, were analyzed. The final sequences were 816–846 nucleotides in length (Table 2). A BLAST search was performed for each sequence in order to locate related sequences. All samples showed a pairwise identity of 98.70–99.50% and coverage 82.40–100% with *H. nana* sequences with accession numbers: LM403673, LM402005, KT951722, and AP017666.

**Table 2:** Genetic sequences from the isolated *H. nana* with variable lengths and GC contents (*pnad5*)

Name	Host location	Host species	%GC	Post-Trim	Length
1MM	Makkah, Saudi Arabia	*Mus musculus*	27.80%	814	834
1MR	Riyadh, Saudi Arabia	*Mus musculus*	28.20%	801	838
2MR	Riyadh, Saudi Arabia	*Mus musculus*	27.60%	800	837
3MM	Makkah, Saudi Arabia	*Mus musculus*	27.70%	798	837
8MM	Makkah, Saudi Arabia	*Mus musculus*	28.20%	803	837
10MR	Riyadh, Saudi Arabia	*Mus musculus*	27.90%	802	834
11MR	Riyadh, Saudi Arabia	*Mus musculus*	27.90%	799	838
12MR	Riyadh, Saudi Arabia	*Mus musculus*	27.80%	802	837
13MM	Makkah, Saudi Arabia	*Mus musculus*	27.60%	799	836
15MR	Riyadh, Saudi Arabia	*Mus musculus*	27.70%	803	838
16MM	Makkah, Saudi Arabia	*Mus musculus*	27.50%	798	816
16MR	Riyadh, Saudi Arabia	*Mus musculus*	27.20%	807	843
19MR	Riyadh, Saudi Arabia	*Mus musculus*	27.80%	802	834
20MR	Riyadh, Saudi Arabia	*Mus musculus*	27.60%	801	837
21MM	Makkah, Saudi Arabia	*Mus musculus*	27.70%	802	839
23RM	Riyadh, Saudi Arabia	*Mus musculus*	28.20%	799	837
25MR	Riyadh, Saudi Arabia	*Mus musculus*	27.50%	803	834
26MR	Riyadh, Saudi Arabia	*Mus musculus*	27.40%	803	819
27MM	Makkah, Saudi Arabia	*Mus musculus*	27.60%	801	836
27MR	Riyadh, Saudi Arabia	*Mus musculus*	27.40%	797	820
32MR	Riyadh, Saudi Arabia	*Mus musculus*	28.10%	802	839
35MR	Riyadh, Saudi Arabia	*Mus musculus*	28.40%	754	846
36MR	Riyadh, Saudi Arabia	*Mus musculus*	28.10%	805	837
37MM	Makkah, Saudi Arabia	*Mus musculus*	28.60%	746	840
40MM	Makkah, Saudi Arabia	*Mus musculus*	27.70%	818	840
40MR	Riyadh, Saudi Arabia	*Mus musculus*	27.50%	806	835
41MR	Riyadh, Saudi Arabia	*Mus musculus*	28.60%	811	844
42MM	Makkah, Saudi Arabia	*Mus musculus*	27.80%	800	837
66RM	Makkah, Saudi Arabia	*Rattus norvegius*	28.00%	801	837
40RM	Makkah, Saudi Arabia	*Rattus norvegius*	27.40%	801	819
22RM	Makkah, Saudi Arabia	*Rattus norvegius*	27.90%	800	838
23RM	Makkah, Saudi Arabia	*Rattus norvegius*	28.2%	799	837

These samples were also similar to the sequence of *H. nana* isolate *y1 pnad5*, partial *cds*, mitochondrial (accession number KT589891), *H. nana* isolate *shz1 pnad5*, partial *cds, mitochondrial* (accession number KT589901), and *H. nana* isolate *s2* gene, partial *cds*, mitochondrial (accession number KT589905) with identity of 98.70–99.40% and coverage of 81–89%.

A multiple sequence alignment was generated for the 31 samples and related sequences using ClustalW algorithm. The sequence LM402005 was set as the reference sequence. The SNPs are shown (Table 3).

**Table 3:** Variations found in the sequences in the present study relative to sequence LM402005

Name	Minimum	Maximum	Length	Change	Coverage	Polymorphism type	Variant frequency
C	5616	5616	1	A -> C	4	SNP (transversion)	25.00%
CA	6442	6443	2	TC->CA	3 -> 4	Substitution	33.3% -> 50.0%
	5634	5634	1	(A)3 -> (A)2	29	Deletion (tandem repeat)	37.90%
C	6439	6439	1	A -> C	5	SNP (transversion)	40.00%
T	6440	6440	1	A -> T	5	SNP (transversion)	40.00%
	5621	5621	1	#NAME?	20	Deletion	45.00%
T	6426	6426	1	A -> T	5	SNP (transversion)	60.00%
	6439	6439	1	#NAME?	5	Deletion	60.00%
	6440	6440	1	#NAME?	5	Deletion	60.00%
	5617	5618	2	(AA)3 -> (AA)2	10	Deletion (tandem repeat)	80.00%
A	5885	5885	1	G -> A	31	SNP (transition)	100.00%
A	5956	5956	1	G -> A	31	SNP (transition)	100.00%
G	6273	6273	1	A -> G	31	SNP (transition)	100.00%
T	6295	6295	1	C -> T	31	SNP (transition)	100.00%
	6427	6432	6	#NAME?	5	Deletion	100.00%
C	6435	6435	1	A -> C	5	SNP (transversion)	100.00%
	6438	6438	1	#NAME?	5	Deletion	100.00%
T	6441	6441	1	C -> T	5	SNP (transition)	100.00%

**Figure 2:**
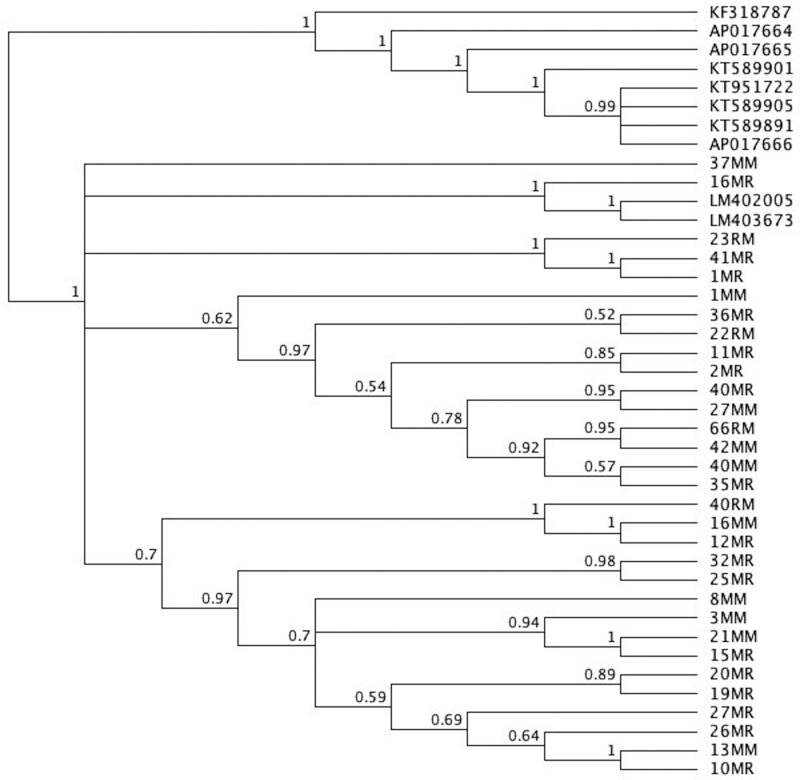
Phylogenetic tree of 31 mice and rats samples in the current study along with similar sequences published in Genbank

MrBayes was used in order to generate the Phylogenetic tree. *Dicrocoelium dendriticum* (accession number KF318787) was used as the outgroup. The Phylogenetic tree with posterior probability values is shown (Figure 2). The sequences placed in a clade of *H. nana* sequences LM402005 and LM403673 (note: the tree is provided Nexus format).

## Discussion

Laboratory animal models, especially rodents of the family, *Muridae*, share important links in food chains in their ecosystems, due to their life style and great biotic potential [17–19]. Compared with most animal species, rodents have a greater ability to harbor many zoonotic agents [20–23]. Due to their broad distribution and close contact with different animals as well as with humans, rodents may act as reservoir hosts for vector-borne disease agents [24]. In conventional animal facilities, rodent colonies either are frequently infected with helminth parasites or become infected in places where they are maintained while waiting to be experimented on [2,25,26]. Based on morphological characters, the Hymenolepidid species that were analyzed had all the characteristic features of genus *Hymenolepis* and were identified as *H. nana*.

Molecular phylogenetic approaches in association with traditional morphological techniques are used extensively for identification, phylogenetic analysis, and differentiation of highly similar Hymenolepidid species infecting laboratory rodents [27–33]. Mitochondria play an essential role in metabolism, apoptosis, illness, and aging [34]. They facilitate oxidative phosphorylation, ATP production and other biochemical functions. Mitochondria contain their own genome, consisting of mitochondrial DNA (mtDNA), often used as a part of molecular phylogenetics studies [35]. Mitochondrial genomes of helminths display unique characteristics such as all genes being coded on the same strand [36].

In the present study, mt genes of *H. nana* were amplified using species-specific primers. A descriptive analysis of *H. nana* mt genes may enable the use of genetic markers in the diagnosis of hymenolepiasis and facilitate epidemiological studies of *H. nana* at a molecular level. Furthermore, the use of mtDNA markers to examine genetic variability in cryptic/sibling species and larval stages of *H. nana* may be vital as morphological descriptions of *H. nana* are still rare [6, 37, 38]. For purposes of the present study, genomic DNA was extracted from 31 specimens of *H. nana*, from two different geographical locations in Saudi Arabia. The lengths of *cox*1 and *pnad5* sequences, obtained separately from the specimens, were 850 bp, and the GC contents of the sequences were 31.6–33.5%, for *cox1*, and 27.2%-28.6% for *pnad5*. The range of genomic similarity determined among specimens by *cox1* primer was 97.1% and by *pnad5* primer was 99.7%.

The inter-specific sequence differences between *cox1* and *pnad5* were found to be low and recorded between *H. nana* (present isolates) and *H. nana* (accession number LM402005, LM403673, KT951722, AP017666, LC063187, GU433102, GU433103, GU433104, AB033412, KT589891, KT589901, and KT589905). These results agreed with those of a previous study that reported lower divergence values between the *Hymenolepis* species that are most related to each other [39].

In the phylogenetic tree, *H. nana* isolates did not exhibit an obvious geographical distinction based on the sequences of the two mtDNA regions. All *H. nana* isolates from Makkah and Riyadh grouped together, indicating that all *H. nana* samples from Makkah and Riyadh were strongly related. Furthermore, isolates from both mice and rats displayed genomic similarity. These results were similar to those of a previous study [14]. Subsequent analyses of genetic sequences of Hymenolepid species have strongly supported monophyly with strong bootstrap values within the cestoda clade. These results substantiated those obtained in previous studies indicating that Hymenolepididae species of the genus *Hymenolepis* may be monophyletic in origin [40–43].

Supported by existing data, the present study, investigated the placement of Hymenolepid species within Hymenolepididae. Results indicated that the present species were deeply embedded in the genus *Hymenolepis* with close relationships to other *Hymenolepis* species, including previously described *H. nana*, as a putative sister taxon. Our results indicate that more indepth phylogenetic studies, which include more taxa and different molecular markers of Hymenolepid species, may be needed in the future. A recent field study provided useful tools for the rapid identification and phylogenetic analysis of Hymenolepidids infecting laboratory rodents. In addition, *cox1* and *pnad5* of *H. nana* that were analyzed by the present study yielded a unique sequence that confirmed their taxonomic position within the family of Hymenolepid species. Also, laboratory rodents should be considered potential natural reservoirs of different parasite species, which require further monitoring in order to improve the awareness of researchers, in order to prevent possible transmission of parasitic zoonosis from laboratory animals.

## Abbreviation

*cox1*: cytochrome oxidase
